# Editorial: New insights into post-translational modification of proteins and immune regulation in carcinogenesis, heart disease, neurodegenerative disorders, and allergic diseases

**DOI:** 10.3389/fphar.2023.1334429

**Published:** 2023-11-30

**Authors:** Zhonghua Li, Liying Ma

**Affiliations:** ^1^ Academy of Chinese Medical Sciences, Henan University of Chinese Medicine, Zhengzhou, China; ^2^ School of Pharmaceutical Sciences, Zhengzhou University, Zhengzhou, China

**Keywords:** post-translational modifications, proteomics, immune regulation, inflammation, inhibitors

In recent years, there has been a growing recognition of the critical role played by post-translational modifications (PTMs) of proteins in various diseases, such as carcinogenesis, heart diseases, neurodegeneration, and allergic diseases. The intricate interplay between PTMs and immune regulation has emerged as a fascinating area of research, offering new insights into the pathogenesis and potential therapeutic targets for human diseases. In this context, we are delighted to present a Research Topic focused on *New Insights into Post-translational Modification of Proteins and Immune Regulation in Carcinogenesis, Heart Disease, Neurodegenerative Disorders, and Allergic Diseases.* This Research Topic of articles aims to provide a comprehensive exploration of the latest advancements in understanding the role of PTMs and immune regulation in these diverse disease contexts.

Our Research Topic begins with an original research paper by Si et al. which provides valuable insights into the role of histone acetylation in ovarian cancer. The authors demonstrate that a novel pyridine derivative, compound H42 effectively inhibits the growth and proliferation of ovarian cancer cells both *in vitro* and *in vivo*, and exhibits inhibitory activity against ovarian cancer progression. Further mechanism study presented that this compound affected the expression of histone acetylase 6 (HDAC6) and the acetylation of *α*-tubulin and heat shock protein 90 (HSP90), and suggest that H42 functions through the regulation of HDAC6-mediated acetylation of HSP90 and *α*-tubulin, leading to downstream effects on cell cycle regulation and cancer cell proliferation. Ovarian cancer is a highly aggressive and difficult-to-treat cancer, and there is a great need for new therapeutic options. Their findings highlight the significance of histone acetylation and HDACs in ovarian cancer progression, and suggest that targeting HDACs with compounds could be a promising therapeutic strategy.

Proteomics, as a robust scientific approach, provides unique insights into the complicated biological processes occurring at the protein level, and serves as an invaluable resource for understanding the complex interplay between proteins and disease, making it an indispensable tool in biomedicine research. Wang et al. utilizes global quantitative phosphoproteomic and proteomic analysis to investigate the molecular mechanism underlying post-infarction chronic heart failure. They identified differentially expressed phosphorylated proteins (DPPs) to elucidate the complex signaling pathways involved in this multifactorial systemic disease. Importantly, their findings highlight the enrichment of DPPs in nucleocytoplasmic transport and mRNA surveillance pathways, providing valuable insights into the dysregulation of these processes in chronic heart diseases. Furthermore, the identification of Bclaf1 Ser658 as a potential therapeutic target and the exploration of predicted upstream kinases offer promising avenues for the development of efficient treatments for post-infarction chronic heart diseases. These findings not only deepen our understanding of the molecular mechanisms underlying heart failure but also pave the way for the development of targeted therapies.

In addition, the review by Liu et al. explores the connection between oxytocin and its receptors (OT/OTR) signaling and bone metabolism. The authors provide a comprehensive review of recent studies that highlight the involvement of OT/OTR in regulating bone marrow mesenchymal stem cells, osteoblasts, osteocytes, chondrocytes, and adipocytes, and these cells are closely associated with post-transcriptional modifications, immunity, and inflammation in the process of bone metabolism. The authors elucidate the molecular mechanisms through which OT/OTR modulate bone formation, resorption, and microstructure, ultimately contributing to the understanding of their anti-osteoporosis effects. It highlights the multifaceted role of OT and OTR in bone metabolism and the potential implications for immune regulation and disease pathogenesis. This article serves as a valuable addition to our Research Topic on PTMs of proteins and immune regulation.

Alzheimer’s disease (AD) is a multifaceted neurodegenerative disorder characterized by cognitive impairment and memory loss. Despite extensive research, successful therapeutic interventions for AD continue to be elusive. In this special Research Topic, Yuan et al. reported their exploration on the neuroprotective properties of Liuwei Dihuang pill (LWD) in the APP/PS1 mouse model of AD. LWD is a traditional Chinese medicine formula, and has long been recognized for its therapeutic benefits in various diseases including neurodegenerative disorders. However, the active constituents and mechanisms of action of LWD have remained largely unexplored until the present. They explored a network pharmacological analysis to predict the potential targets and mechanisms of LWD in AD. And their results showed that LWD improved learning and memory abilities, reduced Aβ deposition, and attenuated neuroinflammation in the hippocampus of APP/PS1 mice. Furthermore, these effects were found to be associated with the autophagy activation pathway regulated by PI3K/Akt signaling pathway and the reduction of inflammation induced by the activation of glial cells. Their research offers new insights into the neuroprotective effects of LWD in AD, and present an additional opportunity of the development of novel clinical treatments. Further research is expected to explore the specific active compounds and their action mechanisms in LWD, which is believed to provide novel drug candidates for AD treatments.

In summary, this Research Topic of research articles provides recent advances in protein post-translational modifications, immune regulation and inflammatory signaling implicated in various disease pathogenesis. And these researches hold significance in advancing our understanding of disease mechanisms and developing novel therapeutic approaches in various pathological conditions. We acknowledge the dedication and scientific rigor of all authors to enrich our understanding of the intricate relationship between PTMs, immune regulation, inflammation and disease pathogenesis.

